# Supplementary material for: Policymakers’ Perspectives Towards Developing a Guideline to Inform Policy on Fetal Alcohol Spectrum Disorder: A Qualitative Study
Source: Int J Environ Res Public Health. 2019 Mar 15;16(6):945. doi: 10.3390/ijerph16060945 (PMC6466131; doi:10.3390/ijerph16060945)
Supplement: Supplementary file 1 [file ijerph-16-00945-s001.pdf]

INTERVIEW GUIDE FOR UNSTRUCTURED INTERVIEWS WITH POLICYMAKERS

Introductions and purpose of interview explained, and consent form signed.

1. Do you have policies/guidelines documents specifically for FASD in your department?
2. Describe which aspects of FASD are the focus on in these policies/guidelines?

Probe on themes:

Department of Health:

- Diagnosis, follow-up services after diagnosis
- Ages and development (what about adolescents and adults)?
- Mental health and psychiatric conditions
- Use of alcohol/substances
- Sexual behavior
- Preventative services = mother alcohol use, health promotion maternal and community education

Department of Education:

- School experience of people with FASD
- Absence from school
- Specific language, cognitive, literacy, social skills or other training? (Child and parents)
- Parental involvement
- Ages and development (what about adolescents)?

Department of Social Development

- Continuing services to people with FASD irrespective of age
- Family services and single parent services
- Living conditions and recreational facilities
- Employment and recreational activities
- Inclusion in communities, schools etc.
- Financial assistance
- Disability services
- Alcohol and substance abuse
- Sexual behavior
- Conflict with law

Preventative services: alcohol abuse of mothers, communities, alcohol as payment, social skills training and family coping with FASD

3. Describe how the policies/guidelines are being implemented? Who is involved in services? Which regions?
4. If the policy/guideline needs to be improved or new policy/guideline needs to be developed, what should be included/excluded?
